# Clinical Profiles Associated with Influenza Disease in the Ferret Model

**DOI:** 10.1371/journal.pone.0058337

**Published:** 2013-03-05

**Authors:** Gregory V. Stark, James P. Long, Diana I. Ortiz, Melicia Gainey, Benjamin A. Carper, Jingyu Feng, Stephen M. Miller, John E. Bigger, Eric M. Vela

**Affiliations:** Biomedical Research Center, Battelle, Columbus, Ohio, United States of America; The Ohio State University, United States of America

## Abstract

Influenza A viruses continue to pose a threat to human health; thus, various vaccines and prophylaxis continue to be developed. Testing of these products requires various animal models including mice, guinea pigs, and ferrets. However, because ferrets are naturally susceptible to infection with human influenza viruses and because the disease state resembles that of human influenza, these animals have been widely used as a model to study influenza virus pathogenesis. In this report, a statistical analysis was performed to evaluate data involving 269 ferrets infected with seasonal influenza, swine influenza, and highly pathogenic avian influenza (HPAI) from 16 different studies over a five year period. The aim of the analyses was to better qualify the ferret model by identifying relationships among important animal model parameters (endpoints) and variables of interest, which include survival, time-to-death, changes in body temperature and weight, and nasal wash samples containing virus, in addition to significant changes from baseline in selected hematology and clinical chemistry parameters. The results demonstrate that a disease clinical profile, consisting of various changes in the biological parameters tested, is associated with various influenza A infections in ferrets. Additionally, the analysis yielded correlates of protection associated with HPAI disease in ferrets. In all, the results from this study further validate the use of the ferret as a model to study influenza A pathology and to evaluate product efficacy.

## Introduction

Influenza A viruses (family *Orthomyxoviridae*) continue to pose a threat to public health and are responsible for an average of 20,000 deaths and 114,000 hospitalizations per year in the United States, and up to 500,000 deaths annually worldwide [1]. The World Health Organization reports that influenza seasons where H3N2 strains are predominate are associated with more severe illness and mortality rates. During the 2008–2009 influenza season, several European countries experienced an outbreak of the H3N2 A/Brisbane/10/07 strain [2]. Additionally, the 2009 human pandemic H1N1 virus was a unique combination of influenza virus genes most closely related to North American swine-lineage H1N1 and Eurasian lineage swine-origin H1N1 influenza viruses [3]. Investigations of initial human cases did not identify exposures to swine and it quickly became apparent that this new virus was circulating among humans and not among U.S. swine herds. Additionally, highly pathogenic avian influenza (HPAI) has caused over 560 human infections characterized by high viral loads, mortality, and fulminate pneumonia [4], [5]. Complications in severe cases included acute respiratory distress syndrome, leukopenia, lymphopenia, hemophagocytosis, multiorgan dysfunction failure, and mortality in approximately 60% of the cases [4], [5]. Because influenza infections continue to be a threat to public health, the availability of animal models is necessary to understand the disease progression, disease clinical profile, and to evaluate vaccines and therapeutic efficacy. Numerous animal species including the mouse, guinea pig, and ferret have been used to study influenza pathogenicity. Because ferrets (*Mustela putorius furo*) are naturally susceptible to infection with human influenza A and B viruses, share similar lung physiology to humans, and the disease state resembles that of human influenza, these animals have been widely used as a model for influenza virus pathogenesis and immunity studies [6]–[8]. Although the ferret model is an accepted model for studying influenza virus pathogenicity and vaccine efficacy [6], [9], a statistical analysis comparing the changes of various biological parameters that can be used to define clinical profiles and predictors of survival/mortality as a result of infection, has not been performed. Here, we report a statistical analysis used to evaluate data involving 269 ferrets infected with seasonal influenza, swine influenza, and HPAI from 16 different studies over a five-year period. The main objective of the study was to compare clinical profiles associated with several influenza strain infections in order to determine whether specific clinical parameters correlate with survival or mortality. Changes associated with body temperature, body weight, activity, viral shedding, and clinical pathology specific to the various influenza viruses were analyzed to describe the clinical profile associated with disease.

## Results

### Assessment of Survival of Ferrets Infected with Influenza Virus

**Figure 1:**
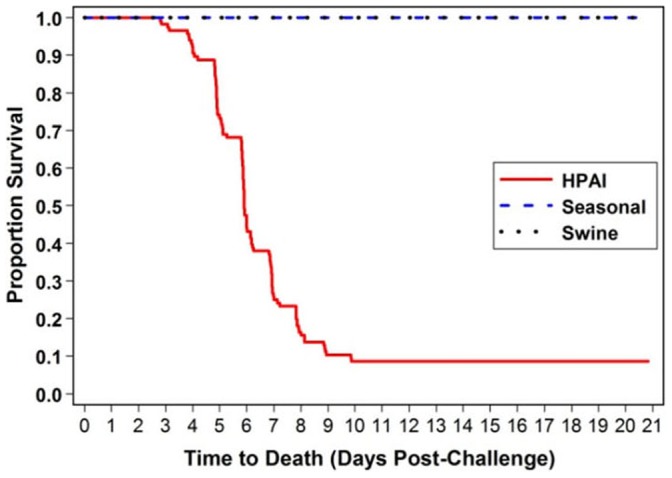
A comparison of survival in ferrets infected with HPAI, seasonal, and swine influenza. Survival data for ferrets challenged with influenza virus were captured in a Kaplan-Meier curve. Mortality was only observed in animals challenged with HPAI.

The ferret model has been established to study numerous viral diseases that cause respiratory complications [6]–[9]. Although the ferret model is an accepted model for studying influenza viruses, the statistical analysis of data from multiple studies which can be used to define clinical profiles and predictors of survival/mortality after influenza infection, has not been performed. Therefore, data from various ferret influenza studies conducted in our laboratory were utilized to delineate a clinical profile associated with HPAI, swine influenza, and seasonal influenza infection. A statistical analysis utilizing only untreated, influenza virus-infected ferrets, was performed to determine correlates of protection by comparing various clinical parameters. Clinical parameters evaluated include survival/mortality, body temperatures, activity, body weights, clinical chemistry parameters, hematology parameters, and viral secretion. Ferrets were infected with 1×10^6^ TCID_50_ of HPAI H5N1 (A/Vn/1203/04), seasonal influenza H3N2 or H1N1 (A/Brisbane/10/07 or A/New Caledonia/20/1999, respectively), or swine influenza (A/California/04/09) by nasal instillation and observed for 14 days. The challenge titer was chosen based on previous natural history studies performed with each of the viruses in the ferret model. On the day of infection, ferrets were anesthetized with Telazol® (16–22 mg/kg, intramuscular) and the infection material was instilled slowly into the nares of the ferrets. Infection with the influenza viruses led to various clinical parameters that could be associated with these viruses. Infection with H5N1 A/Vn/1203/04 resulted in measureable disease and 93% mortality by day 10 post-challenge (Fig. 1). Any ferret surviving through 10 days post-infection fully recovered from the disease. In contrast, seasonal influenza and swine influenza challenge in ferrets did not result in lethality, but did result in a measureable disease state.

### Weight, Body Temperatures, and Activity Changes Associated with Influenza Virus Infection

**Figure 2:**
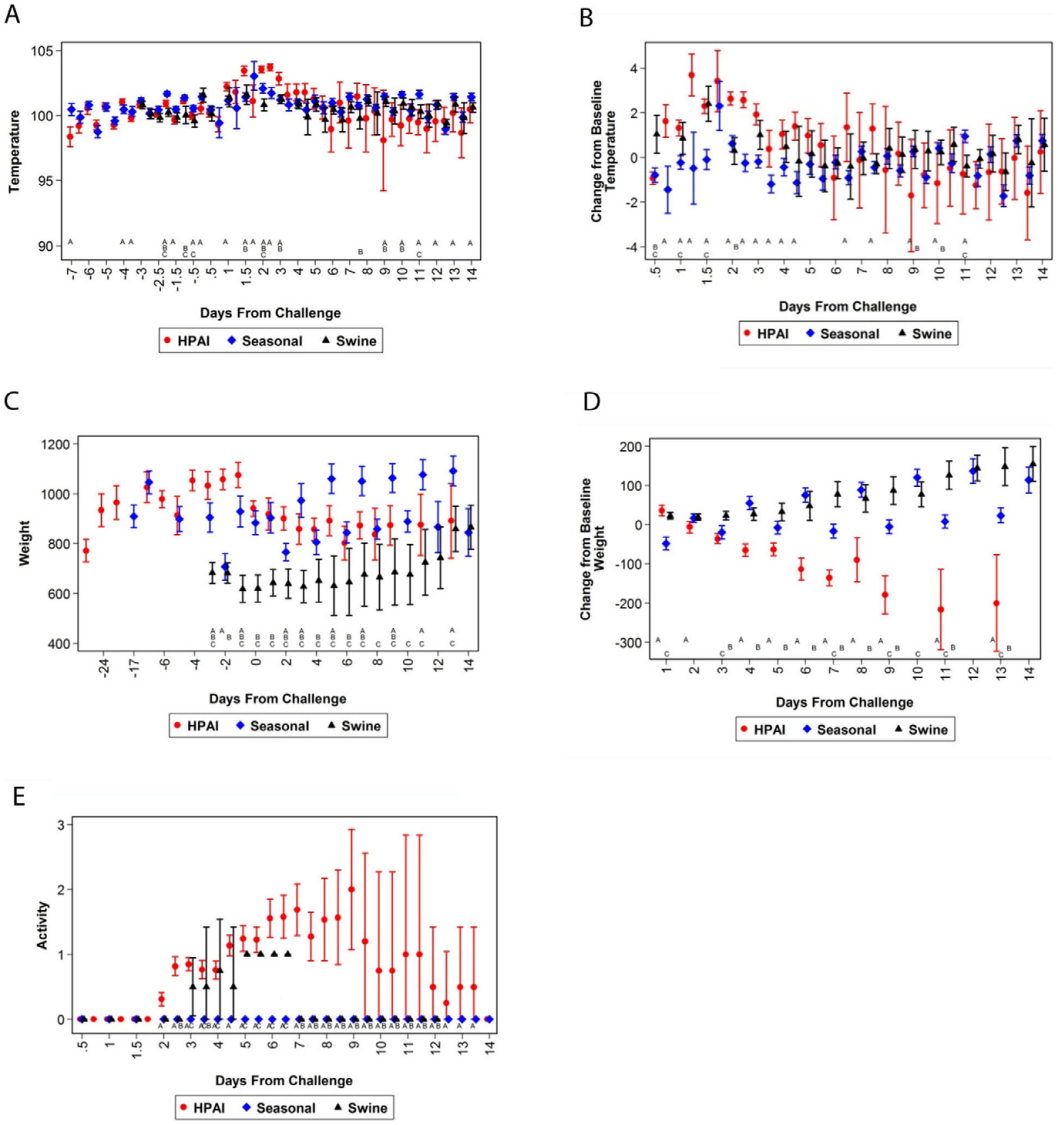
A comparison of temperature, weight, and activity of influenza-infected ferrets. A statistical comparison was performed on ferrets infected with HPAI, seasonal, or swine influenza virus: (A) temperature, (B) change in temperature from baseline, (C) weight, (D) change in temperature from baseline, and (E) activity. A represents a significant difference when comparing HPAI and seasonal influenza; B represents a significant difference when comparing HPAI and swine influenza; and C represents a significant difference when comparing swine influenza and seasonal influenza. The error bars represent the 95% confidence intervals.

Influenza virus infection in ferrets, regardless of the strain, resulted in a disruption of their normal diurnal temperature fluctuations and an increase in temperature that could be observed within 24 hours post-challenge (Fig. 2A). Statistical analyses performed on the absolute temperatures and temperature changes from baseline (Fig. 2A and 2B) suggest that HPAI-infected ferrets experienced significant variations in body temperatures and the fever intensity on various days post-infection when compared to ferrets infected with seasonal- or swine influenza viruses. For instance, animals infected with HPAI exhibited more severe fever, as demonstrated by comparing changes to baseline values. Additionally, infection with HPAI resulted in a longer duration of fever when compared to seasonal and swine influenza-infected animals. Surviving animals infected with influenza virus, regardless of strain, exhibited normal diurnal temperature patterns approximately 9 to 10 days post-challenge. These animals remained relatively normal throughout the remainder of the studies.

Weight loss is a clinical profile associated with HPAI infection in ferrets. Ferrets infected with HPAI lost a significant amount of weight starting two days post-infection (Fig. 2C and 2D). Statistical analysis demonstrates a significant difference in the amount of weight that was lost in animals infected with HPAI when compared to those infected with seasonal or swine influenza. In surviving animals infected with HPAI, the weight appeared to stabilize nine days post-challenge. In contrast, ferrets challenged with seasonal and swine influenza did not experience significant weight loss; moreover, in most cases, the weight remained constant or weight gain was observed following infected. Overall, most of the weight loss was associated with HPAI-infected ferrets, which is likely attributed to general malaise and inappetance. Malaise and inappetance is more pronounced in ferrets challenged with HPAI when compared with seasonal or swine influenza (data not shown). Thus, weight loss is a clinical profile associated with HPAI and may be used as a differentiator when comparing infection of various types of influenza virus.

The animal activity was also observed following infection and used to evaluate the clinical profiles associated with HPAI, seasonal, or swine influenza (Fig. 2E). Activity scores were based on the scoring system described by Reuman *et al.* [10] and Zitzow *et al* [8] in which normal animals exhibiting normal, alert behaviors receive a “0” while animals that are neither alert nor playful receive a score of “3.” Animals infected with HPAI began to demonstrate abnormal activity approximately two days post-challenge. Typically, ferrets challenged with HPAI became less active following infection, as indicated in the activity scores. Surviving HPAI-infected animals demonstrated lower activity on days 9–10 post-infection; however, most received normal activity scores 12 to 14 days post-infection. In contrast, animals infected with seasonal or swine influenza did not receive similar activity scores indicating less activity. These animals received highest scores 4 to 6 days post-infection. However, these scores rarely progressed to a “2” indicating that these ferrets were still relatively normal and active only when stimulated. Animals infected with seasonal influenza virus did not demonstrate any changes in activity. In all, low activity is associated with HPAI infection (indicative of a higher activity score) and any animal receiving a score of “3” did not survive infection.

### Influenza Virus Secretions Isolated from Ferret Nares

**Figure 3:**
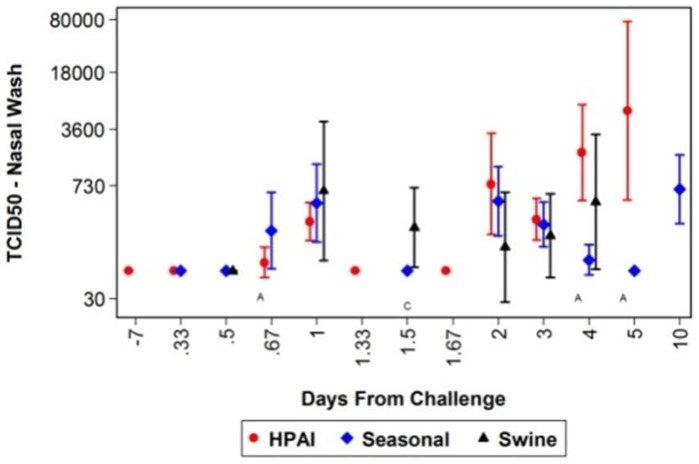
Secretion of virus isolated from nasal washes. A standard TCID50 assay was performed on collected nasal wash samples on various days post-challenge. The CPE on MDCK cell monolayers were scored and the TCID_50_ was calculated using the Spearman Kärber method. Geometric means and 95% confidence intervals were plotted.

Influenza virus-infected ferrets secrete or sequester measurable virus from their nares. Thus, a statistical analysis was performed to determine whether differences in virus secretion could be used as a clinical profile of influenza disease in ferrets (Fig. 3). In all, animals infected with HPAI secreted a higher virus titer than animals infected with seasonal or swine influenza. When comparing virus secretions from ferrets infected with HPAI and seasonal influenza, statistically significant differences were observed on days 4 and 5 post-infection. However, no statistically significant differences were observed when comparing animals infected with HPAI and swine influenza. Unfortunately, day four post-infection data from ferrets infected with swine influenza was not available for comparison against HPAI-infected ferrets, which showed high virus titers on that day.

### Clinical Pathology Changes Associated with Influenza Virus Challenge

**Figure 4:**
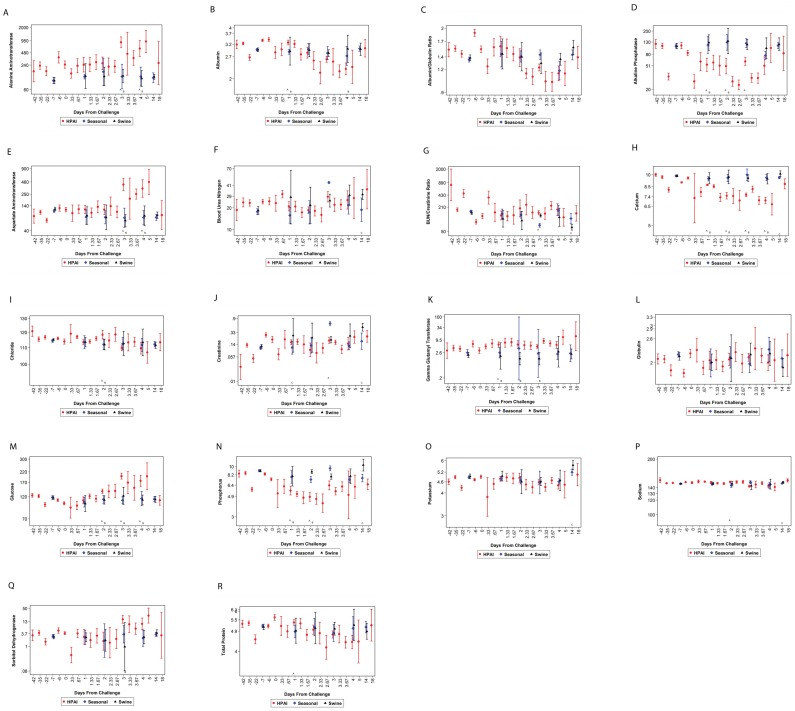
A comparison of clinical chemistry parameters of influenza-infected ferrets. Blood was collected into SST tubes and clinical chemistry analysis was conducted on the Advia 1200 (Siemens). A comparison of the following clinical chemistry parameters was performed on ferrets infected with HPAI, seasonal, or swine influenza virus: (A) alanine aminotransferase (ALT), (B) albumin, (C) albumin/globulin ratio, (D) alkaline phsophatase, (E) aspartate aminotransferase (AST), (F) blood urea nitrogen (BUN), (G) BUN/creatinine ratio, (H) calcium, (I) chloride, (J) creatinine, (K) gamma glutamyl transferase (GGT), (L) globulin, (M) glucose, (N) phosphorus, (O) potassium, (P) sodium, (Q) SDH, and (R) total protein. A represents a significant difference when comparing HPAI and seasonal influenza; B represents a significant difference when comparing HPAI and swine influenza; and C represents a significant difference when comparing swine influenza and seasonal influenza. Geometric means and 95% confidence intervals (error bars) were plotted.

Specific clinical pathology changes are hallmarks of HPAI infection in humans [11]–[14]. Thus, multiple hematology and clinical chemistry parameters were evaluated to further characterize the clinical profile of ferrets infected with influenza. HPAI infection in ferrets resulted in changes to several clinical chemistry parameters (Fig. 4A-R). Increases in alanine aminotransferase (ALT), aspartate aminotransferase (AST), and sorbitol dehydrogenase (SDH), and the decrease observed in albumin are all indicative of liver disease. Increases in these parameters were more profound in animals infected with HPAI. When compared to animals infected with either seasonal or swine influenza, statistical differences were observed for ALT, AST, and SDH on specific days. Additional significant differences for specific days post-infection were observed for other clinical chemistry parameters when comparing the results obtained in animals infected with HPAI, seasonal, or swine influenza including changes in albumin, creatinine, chloride, calcium, glucose, phosphorus, potassium, and total protein. Hypocalcemia was observed in ferrets infected with HPAI, while calcium levels remained constant in animals infected with the seasonal and swine flu strains. These results may be indicative of kidney failure or insufficient total protein in the blood, which was observed in HPAI-infected animals. Additionally, creatinine levels can be used as a biomarker for renal health and changes in creatinine levels may indicate changes to overall kidney health and function. Altogether, changes in calcium, creatinine, and total protein suggest decreased kidney function in animals infected with HPAI. The differences observed in the blood urea nitrogen (BUN), BUN/creatinine ratio, and chloride levels may also be attributed to the decreased kidney function. A decrease in albumin levels can be observed in animals infected with HPAI while globulin levels remain relatively constant. This results in changes in the albumin/globulin ratio. Though no albumin/globulin ratio differences were observed when comparing influenza infection in ferrets, a marked decrease in the ratio occurred post-infection, which may be indicative of an overproduction of globulins (Fig. 4C). Cholestasis and decreased protein intake and synthesis (indicative by the clinical pathology panel) may also attribute in decreased albumin/globulin. HPAI-infected animals experienced low alkaline phosphatase levels. This result is not surprising considering the animals were likely experiencing hypophosphatasia. The elevated glucose levels in the HPAI-infected animals is also expected considering that HPAI-infected animals were showing more severe signs of disease when compared to the animals infected with seasonal or swine influenza virus. Gamma glutamyl transferase (GGT) levels can be used as a biomarker for liver and pancreas disease. The GGT levels in ferrets infected with HPAI slightly rose after infection and the levels were elevated when compared to animals infected with seasonal or swine influenza. However, it should be noted that the elevated levels were only marginally elevated and more data needs to be analyzed to determine whether these elevated levels are significantly elevated during the overall disease process.

**Figure 5:**
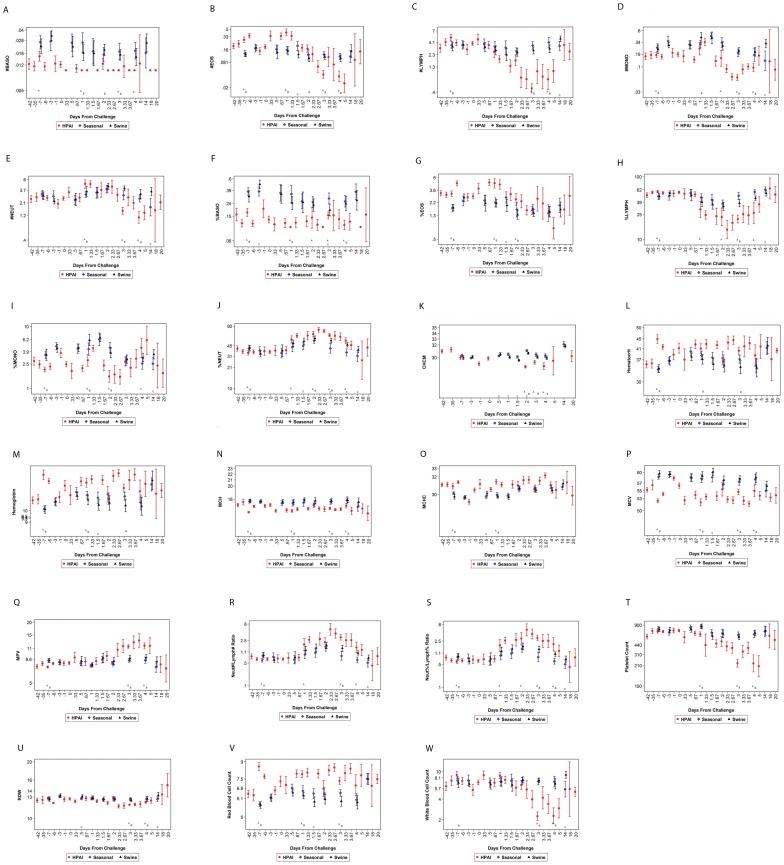
A comparison of hematology parameters of influenza-infected ferrets. Blood was collected into EDTA tubes and hematology analysis was conducted on the Advia 120 (Siemens). A comparison of the following hematology parameters was performed on ferrets infected with HPAI, seasonal, or swine influenza virus: (A) number of basophils, (B) number of eosinophils, (C) number of lymphocytes, (D) number of monocytes, (E) number of neutrophils, (F) percentage of basophils;, (G) percentage of eosinophils, (H) percentage of lymphocytes, (I) percentage of monocytes, (J) percentage of neutrohils, (K) CHCM (cell HGB concentration mean), (L) hematocrit, (M) hemoglobin, (N) MCH (mean corpuscular hemoglobin), (O) MCHC (mean corpuscular hemoglobin concentration), (P) MCV (mean corpuscular volume), (Q) MPV (mean platelet volume), (R) number of neutrophil/number of lymphocyte ratio, (S) percentage of neutrophil/percentage of lymphocyte ratio, (T) platelet count, (U) RDW (red cell distribution width), (V) RBC (red blood cell) count, and (W) WBC (white blood cell) count. A represents a significant difference when comparing HPAI and seasonal influenza; B represents a significant difference when comparing HPAI and swine influenza; and C represents a significant difference when comparing swine influenza and seasonal influenza. Geometric means and 95% confidence intervals (error bars) were plotted.

Additionally, several changes were observed in the hematology parameters as a result of influenza virus infection. Thrombocytopenia, leucopenia, and lymphopenia are classical “hallmarks” of HPAI infection in humans. Ferrets also experience thrombocytopenia (Fig. 5T), leukopenia (Fig. 5W), and lymphopenia (Fig. 5C) when infected with HPAI (Fig. 5). These three hematology parameters were mainly observed in ferrets infected with HPAI, and thus may be considered as part of the clinical profile associated with HPAI infection. It is interesting that seasonal and swine influenza led to increased basophil numbers and the percentage of basophil granolucytes. Increased basophil numbers can be observed during various viral infections and indicate some level of an inflammatory response. The significant increases in hematocrit (HCT) and red blood cells (RBC) may signify hypoxia in the HPAI-infected animals, in addition to decreased hydration and secondary polycythemia. HCT and RBC increases are more extreme in HPAI-infected ferrets when compared to ferrets infected with seasonal or swine influenza virus. These increases are likely the result of decreased intake of fluids. Additionally, the increased RBC levels may explain the elevated hemoglobin levels and the differences observed in mean corpuscular hemoglobin concentration (MCHC) and mean corpuscular hemoglobin (MCH) levels in HPAI-infected ferrets. The mean platelet volume (MPV) was elevated 2–5 days post HPAI infection, which is likely caused by the observed thrombocytopenia. Reductions in the numbers of eosinophils, monocytes, and neutrophils were also mainly associated with HPAI infection in ferrets. Changes in red cell distribution width (RBC) were observed when comparing the animals infected with the different viral strains. This may be due to deficiencies in iron or vitamin 12, which is consistent with the fact that infected animals are routinely malnourished due to inappetance. The elevated Neut#/Lymph# and Neut%/Lymph% ratio observed in infected animals are in response to HPAI and are noticeably elevated when compared to animals infected with seasonal and swine influenza. In all, the clinical pathology changes observed in influenza virus-infected ferrets is similar to that observed in humans, which translates into these specific parameters as appropriate clinical profiles of influenza virus disease. Additionally, the changes associated with increased AST, ALT, SDH, eosinopenia, monocytopenia, neutropenia, in addition to the observed thrombocytopenia, leukopenia, and lymphopenia are associated with HPAI infection when comparing to seasonal and swine influenza, and thus may be considered as part of the clinical profile associated with HPAI.

## Discussion

Although the ferret model is an accepted model for studying influenza virus pathogenicity and vaccine efficacy [6], [9], a statistical analysis comparing the changes of various biological parameters that can be used to define clinical profiles and predictors of survival/mortality as a result of infection, has not been published in the literature. We compared data from 269 male ferrets from 16 studies over a five-year period to evaluate changes in various biological parameters associated with influenza disease with the aim of describing a clinical profile and correlates of protection between three distinct influenza viruses. High mortality (approximately 93%) was associated with ferrets infected with HPAI H5N1 A/Vn/1203/04. Other studies performed in our laboratory have demonstrated various mortality rates associated with other HPAI viral strains, but the highest mortality rates have been associated with H5N1 A/Vn/1203/04 (data not shown). We did not observe mortality in ferrets challenged with various seasonal or swine influenza viruses. The difference in virulence may be associated with various viral or host system factors. For instance, different levels of viremia and the amount of viral replication within the specific host animal tissues may be a factor. A/Vn/1203/04 has been shown to have a lower infection 8 hours post-infection when compared to other influenza virus strains [11]. However, H5N1 A/Vn/1203/04 replicated to titers similar to those of the other influenza virus strains 24 hours post-infection and induced higher levels of cell necrosis when compared to other influenza viruses [11]. This published report also demonstrates a unique ability to recover H5N1 A/Vn/1203/04 from both the apical and basolateral surfaces of cells, which may be indicative of a virulence factor that is associated with this virus [11]. Elevated mortality rates have also been correlated with elevated viral replication and viral load, resulting in extreme cytokine production [12]. Moreover, H5N1 A/Vn/1203/04 has been shown to induce elevated levels of proinflammatory cytokines when compared to other influenza viruses [11]. Additionally, the presence of the proper influenza virus receptors on host cells and the specificity of the various influenza viruses to these receptors may impact virulence [13], [14]. All of these factors likely contribute the virulence of A/Vn/1203/04 when compared to other viruses.

Data from our study suggest that statistical differences in several clinical parameters occur when comparing ferrets infected with HPAI, seasonal, or swine influenza viruses. For instance, HPAI-infected animals experience higher body temperatures, a long duration in fever, greater weight loss, less activity, and increased viral shedding. Additionally, this study demonstrates that temperature increases, thrombocytopenia, leukopenia, and lymphopenia are not only hallmarks of disease in humans [9], [11]–[14], but may also be clinical profiles associated with disease in ferrets. Additionally, elevated AST, ALT, SDH, along with a decrease in eosinophil, monocyte, and neutrophil counts, as well as the observed thrombocytopenia, leukopenia, hypocalcemia, and lymphopenia can be used delineate the clinical profile for HPAI infection in ferrets. Changes in creatinine, total protein, BUN, chloride, and hypocalcemia suggest potential kidney damage in HPAI-infected ferrets. When comparing these levels to ferrets infected with seasonal or swine influenza, it appears that HPAI infection leads to more liver dysfunction. Thus, renal failure or decreased kidney function also appears to be biomarker of HPAI disease and may lead increased mortality when compared to the other influenza viruses tested. Additionally, changes observed to various clinical pathology profiles suggest HPAI infection in ferrets may lead to more severe anemia when compared to ferrets infected with seasonal or swine influenza viruses. Elevated RBC and HCT, hepatocyte damage, characterized by elevated AST, ALT, and SDH, a decrease in activity, fever, and weight loss are all clinical profiles associated with influenza disease. Thus, it appears that a clinical profile or disease signature is specific to HPAI, seasonal, or swine influenza infection. Additionally, evaluating such parameters on specific days post-infection, leads to a further characterized disease state in which HPAI, seasonal, and swine influenza disease appear to have a distinct disease signature. Furthermore, when evaluating mortality and survival associated with HPAI infection, specific clinical profile parameters can be used as correlates of protection (Table 1). A statistical analyses of HPAI-infected ferret data collected over a 5 year period demonstrates that increased thrombocytes and lymphocytes, three days post-challenge, may be used to predict whether an animal will survive. Thus, the data suggest that the total number of thrombocytes and lymphocytes influence survival when ferrets are infected with HPAI. Moreover, maintaining body weight and mean corpuscular volume [(MCV) average RBC volume], three days post-infection, may lead to a greater chance of survival (Table 1). HPAI-infected animals show signs of anorexia and begin to lose weight. However, the data suggest that maintaining body weight correlates with survival of the animal, which is a typical correlation associated with most disease states. Statistical analyses of these parameters show significant correlation with survival in the HPAI survival model since the positive slope estimate suggests that if there is a smaller decrease from baseline for a representative parameter, then the animal has a greater probability of surviving infection.

**Table 1:** Correlates of Survival in HPAI-Infected Ferrets.

Parameter	Days of Post-Challenge	Slope	P-Value
Decrease in Lymphocytes	3	3.65	0.04
Change in MCV	3	97.7	0.04
Decrease in PLT	3	20.7	0.02
Decrease in Body Weight	5	0.014	0.03

Severe human disease due to influenza virus infection results in lymphopenia, leucopenia, fever, anemia, and changes in clinical pathology. Complete analyses of clinical chemistry profiles in humans infected with swine influenza have been described [15] and the observed changes in this current study add credence to the ferret model. Though many changes to the host system, as a result of influenza virus infection, have been observed, a statistical comparison using data from multiple studies on signs of disease and changes associated with body temperature, clinical pathology, and virus shedding has not been reported. Thus, this study aimed to describe the clinical profile associated with disease in ferrets caused by the various influenza viruses and to determine whether correlates of protection could be identified. The identification of such correlates of protection could shape the strategic targets of novel therapeutics and prophylaxis. For example, statistical analyses of the data presented here suggest that the survival of ferrets infected with various influenza viruses exhibited greater lymphocyte numbers, less change in MCV, higher platelet numbers, and overall higher body weights. Individually, these parameters may not affect the survival of a ferret infected with influenza viruses. However, collectively, these parameters have been statistically identified as correlates of protection and thus, may represent targets for novel therapeutics and prophylaxis.

In conclusion, establishing the clinical profile or disease signature associated with influenza disease is necessary to establish correlates of protection. Previous published work has suggested that virulence factors and mortality associated with various influenza viruses may correlate with several host and viral factors including: the presence of influenza receptors on cells; temperature changes; induction of cellular necrosis; viremia and viral titers in host tissues; and the induction of a severe immune response [12]–[14]. Our study suggests that the severity and duration of febrile temperatures, overall lymphocyte and platelet numbers, changes in MCV, and overall body weight associated with the host animal after influenza infection may also have a role in the pathogenesis and disease state, which may serve as correlates of clinical disease in ferrts. Furthermore, delineation of the influenza clinical profile can be used to establish proper endpoints in studies designed to test vaccine or therapeutic/prophylaxis efficacy. In all, a statistical analysis of influenza data from multiple studies suggest clinical parameters that correlate to HPAI disease in ferrets and helps validate the use of ferrets as a model system to study influenza pathogenesis and evaluate product efficacy.

## Materials and Methods

### Ethics Statement

All experiments were conducted according to protocols reviewed and approved by the Battelle Biosafety Committee and the Institutional Animal Care and Use Committee of Battelle which adhere to the National Institutes of Health guidelines for the care and use of laboratory animals. Influenza-infected animals were housed in an ABSL-3 enhanced facility.

### Animal Population and Selection Criteria

In order to delineate a clinical profile associated with HPAI, swine influenza, and seasonal influenza infection, data from various ferret influenza studies conducted at the Battelle Memorial Institute were utilized. These studies were originally designed to evaluate several vaccines and therapeutics against the aforementioned influenza viruses. All ferrets were purchased from Triple F Farms (Sayre, PA) and were 8–15 weeks of age upon arrival (total of 269 animals). The animals were confirmed to be seronegative by hemagglutination inhibition assay (HAI) for the currently circulating influenza viruses and A/VN/1203/04. Two to three ferrets were socially housed for the duration of the studies. Prior to the initiation of the studies, all ferrets were evaluated by a licensed veterinarian and determined to be in good health and free from malformations and signs of clinical disease. During the 7–10 day quarantine period, animals were randomized by weight into groups. Ferrets were infected with 1×10^6^ TCID_50_ (high dose), 1×10^6^ TCID_50_ of A/Brisbane/10/07 (H3N2), or 1×10^6^ TCID_50_ of A/California/04/09 (H1N1). In total, analyses was conducted on 128 animals challenged with HPAI (9 studies), 90 animals challenged with seasonal influenza virus (3 studies), and 30 animals challenged with swine influenza virus (3 studies).

Any animal meeting the following criteria were euthanized:

- Presence of seizures
- Limb paralysis which prevents the animal from obtaining food and water
- Respiratory distress
- Unresponsive to touch or external stimuli
- Moribundity.

Animals were sedated prior to administration of an overdose of euthanasia agent containing pentobarbital. Discomfort and distress were limited to that which was unavoidable in the conductance of the study. All study procedures were approved in accordance to the guidelines set by the Institutional Animal Care and Use Committee. All work involving infected animals or virus was performed in the Biosafety level (BSL)-3 laboratory.

### Intranasal Inoculation

On the day of challenge (Study Day 0), animals were anesthetized with Telazol® (16–22 mg/kg, intramuscular) and the challenge material was instilled slowly into the nares of the ferrets using a micropipettor, alternating sides during the instillation. Influenza challenge stocks were prepared in specific pathogen free embryonated chicken eggs and tested for sterility, *Mycoplasma*, and hemagglutinin sequence confirmation. Challenge material was diluted in calcium- and magnesium-free phosphate buffered saline (CMF-PBS) to a target dose delivered in 0.6 mL. A portion of the diluted challenge material was analyzed by Median Tissue Culture Infectious Dose assay (TCID_50_) to confirm viral dose.

### Temperature Measurements

Animals were implanted with programmable temperature transponder chips for monitoring body temperature prior to challenge. Temperature transponder chips (2 per animal) were implanted in the shoulder and rump area (1 per region). Two transponders were implanted in the event that one of the transponders was to fail during the course of the studies. Temperature readings were taken from both the rump and the shoulder in the morning and the evening. These temperature values were then averaged for all statistical analyses for each animal at each time point. Similarly, baseline temperatures for each ferret were calculated by averaging morning and afternoon temperatures for seven days prior to challenge.

### Body Weight Measurements

Animals were weighed during quarantine for randomization, on the day of challenge (Study Day 0) for the baseline body weight, and then daily through the end of each study. An additional weight at termination was not collected if the weight had already been taken that day.

### Clinical Scores

Observations were recorded twice daily from the time the animals arrived until challenge [9]. Following challenge and until the end of each study, clinical observations were recorded twice daily. The following scoring system based on that described by Reuman *et al.* 1989; see also Zitzow *et al*. 2002 [8], [10].

0 = alert and playful

1 = alert but playful only when stimulated

2 = alert but not playful when stimulated

3 = neither alert nor playful when stimulated.

### Blood Collections

Blood samples were collected from the anterior vena cava on anesthetized ferrets. Any animal found moribund underwent a terminal bleed prior to euthanasia. A sample was not collected on animals found dead. At the end of each study, surviving animals were euthanized following a terminal bleed.

### Clinical Pathology

For hematology analysis on the ADVIA® 120 (Siemens, Tarrytown, NY), blood was collected into EDTA tubes according to Table 1. For clinical chemistry analysis conducted on the ADVIA® 1200 (Siemens, Tarrytown, NY), blood was collected into SST tubes according to Table 1. The hematology parameters analyzed included: Red Blood Cell Count (RBC, 10^6^ cells/µL), Hemoglobin (HGB, g/dL), Hematocrit (HCT, %), Mean Corpuscular Volume (MCV, fL), Mean Corpuscular Hemoglobin (MCH, pg), Mean Corpuscular Hemoglobin Concentration (MCHC, g/dL), Cell Hemoglobin Concentration Mean (CHCM, g/dL), Red Cell Distribution Width (RDW, %), Platelet Count (PLT, 10^3^ cells/µL), Mean Platelet Volume (MPV, fL), White Blood Cell Count (WBC, 10^3^ cells/µL), Neutrophils (10^3^ cells/µL), Lymphocytes (10^3^ cells/µL), Neutrophils/Lymphocytes Ratio (N/L Ratio), Monocytes (10^3^ cells/µL), Eosinophils (10^3^ cells/µL), Basophils (10^3^ cells/µL) and Large Unstained Cells (LUC, 10^3^ cells/µL). The clinical chemistry parameters analyzed included: Total Bilirubin (mg/dL), Aspartate Aminotransferase (AST, U/L), Alanine Aminotransferase (ALT, U/L), Sorbitol Dehydrogenase (SDH, U/L), Glucose (mg/dL), Alkaline Phosphatase (ALP, U/L), Gamma Glutamyl Transferase (GGT, U/L), Total Protein (g/dL), Albumin (g/dL), Globulin (g/dL), Albumin/Globulin Ratio (A/G Ratio), Blood Urea Nitrogen (BUN, mg/dL), Creatinine (mg/dL), BUN/Creatinine Ratio, Sodium (mEq/L), Potassium (mEq/L), Chloride (mEq/L), Calcium (mg/dL), and Phosphorus (mg/dL).

### Hemagglutination Inhibition Assay (HAI)

Serum samples were initially treated with receptor destroying enzyme (RDE) to inactivate non-specific inhibitors prior to HAI assay. A standard HAI assay [15] using chicken erythrocytes with seasonal influenza strains or horse erythrocytes with HPAI strains was used to screen for previous exposure in pre-study uninfected animals.

### Hemagglutination Inhibition Assay (HAI) and Median Tissue Culture Infectious Dose (TCID_50_)

A standard HAI assay using chicken erythrocytes with seasonal influenza strains or horse erythrocytes with HPAI strains was used to screen for previous exposure in pre-study uninfected animals [15]. A standard TCID_50_ assay using MDCK cells was used to determine virus titer of tissue samples [16]. The presence of viral infection was determined by an *in situ* influenza A anti-nucleoprotein ELISA [15] and the titer was determined by the Spearman-Kärber method [17].

### Statistical Analyses

Analysis of variance models were fitted to the data from each parameter at each time point. Hematology, clinical chemistry, CRP, and TCID_50_ data were log-transformed for analysis. These models were also fitted to the change from baseline data for each parameter except CRP, and TCID_50_. Baseline was defined as the last measured value before infection. The models were used to test for significant difference between each pair of parameters in the data or change from baseline data using a Bonferroni adjustment for the number of comparisons. Plots were produced showing means (weight, temperature, and activity) or geometric means (hematology, clinical chemistry, TCID_50_) and 95% confidence intervals. These plots also indicate which comparisons were significantly different.

Kaplan-Meier estimates were plotted and a log-rank test was performed to compare survival rates among the influenza virus-infected ferrets. The SAS® MULTTEST procedure was used to adjust for multiple comparisons at the 0.05 level of significance using the Bonferroni-Holm method. Logistic regression models were fit to the change from baseline parameters to determine which variables were significantly associated with survival. These models included HPAI infected ferrets only because mortality was not observed in either of the other strains.
